# Meat quality, fatty acids, volatile compounds, and antioxidant properties of lambs fed pasture versus mixed diet

**DOI:** 10.1002/fsn3.1039

**Published:** 2019-08-02

**Authors:** Yulong Luo, Bohui Wang, Chang Liu, Rina Su, Yanru Hou, Duo Yao, Lihua Zhao, Lin Su, Ye Jin

**Affiliations:** ^1^ College of Food Science and Engineering Inner Mongolia Agricultural University Hohhot China

**Keywords:** antioxidant status, feeding regimens, lamb, fatty acid, volatile compounds

## Abstract

The aim of the present work was to investigate the effects of feeding regimens (pasture vs. mixed diet) on meat quality, fatty acids, volatile compounds, and antioxidant properties in lamb meat. In total, 24 lambs were allotted into two feeding regimens at 10.23 kg live weight. Lambs were fed on pasture grass (PG group, *n* = 12) or mixed diet (M group, *n* = 12). *Longissimus* *thoracis* (LT) muscle samples from the M group had a higher intramuscular fat (IMF) (*p* < 0.05), pH_45min_value (*p* < 0.01), and ash (*p* < 0.05) than the PG group. In contrast, the shear force (*p* < 0.05), L*(*p* < 0.05), and b* (*p* < 0.001) in M group were lower than in PG group. Analyses indicated that PG group contained higher linolenic acid (C18:3n3) and docosatrienoic acid (C22:3n6) (*p* < 0.05) than the M group. Major volatile compounds in the muscles included hexanal, heptanal, nonanal, octanal, 1‐pentanol, 1‐hexanol, 1‐octen‐3‐ol, and 2,3‐octanedione. The levels of hexanal, nonanal, and 2,3‐octanedione were significantly lower in PG lamb muscle (*p* < 0.01). In contrast, 1‐pentanol and 1‐hexanol levels were higher in M lamb muscle (*p* < 0.01). Muscle from PG lamb exhibited higher catalase (CAT) and glutathione peroxidase (GPx) activity (*p* < 0.05). PG muscle also contained a higher radical‐scavenging ability (RSA; *p* < 0.001) and cupric‐reducing antioxidant capacity (CUPRAC; *p* < 0.05). Overall, the improved antioxidant status in PG muscle inhibited lipid peroxidation (aldehydes and ketones), thereby improving the meat quality.

## 1 INTRODUCTION

Mongolian lamb from Inner Mongolia is a popular mainstay animal product that has been bred through long‐term natural selection. The meat is considered to have a high nutritional value with favorable fatty acid content (Luo et al., 2015). Confined feeding of animals in pens or barns is becoming increasingly popular in China, especially with the increasing demand for meat and encroaching urban sprawl. Feedlot or housed animal feeding, especially in China, is now being used to maintain an ecological balance and to halt the destruction of the Mongolian grassland. Additional benefits for confined or feedlot feeding of animals include economics due to an increase in weight gain (Crane et al., 2017), but meat quality can also be affected by the feeding regimen (Duckett, Neel, Lewis, Fontenot, & Clapham, 2013; Lobón et al., 2017). For example, Enser et al. (1998) reported that lambs fed mixed diet (M) have higher levels of lipid and monounsaturated fatty acids in their muscle, while lambs fed pasture grass (PG) contain higher levels of polyunsaturated fatty acids (PUFAs) including arachidonic acid, docosahexaenoic acid (DHA), and eicosapentaenoic acid (EPA). The latter PUFAs are recognized as being highly beneficial to the human diet (Ruiz, Vazquez, & Campos, 2017). However, lipids, especially unsaturated fatty acids, are prone to oxidization and may act as pro‐oxidants resulting in the development of off‐flavors (Kolakawska & Bartosz, 2014).

Regarding meat quality, volatile compounds play a key role in meat's sensory attributes. Volatile compounds are also considered to be indicators of oxidative stability. For example, aldehydes and ketones resulting from fatty acid oxidation are known to contribute to rancidity (Descalzo et al., 2007). Overall, the oxidative stability of meat depends on a balance between antioxidant and pro‐oxidant components. Daley, Abbott, Doyle, Nader, and Larson (2010) reported that grass‐fed beef exhibit lower oxidative deterioration in their muscle compared with grain‐fed beef. This difference may be due to the relatively high content of antioxidant compounds found in forage compared to grain.

In the present study, the effects of two feeding regimens (pasture grass fed vs. a mixed diet fed) were investigated with respect to possible changes in meat quality. Quality parameters that were investigated included the types and levels of fatty acids (FA), volatile compounds, and antioxidant properties.

## 2 MATERIAL AND METHODS

### 2.1 Feeding regimens

Twenty‐four Mongolian lambs with an initial body weight of 10.23 ± 0.79 kg and 3 months of age were used in a completely randomized design for a 6‐month feeding period preceded by a 7‐day adaptation. The animal experiments were approved by the Committee of Animal Experimentation and were performed under the institutional guidelines for animal experiments of the College of Animal Science, Inner Mongolian Agricultural University, China. The experiment was performed according to recommendations proposed by the European Commission (1997) to minimize the suffering of animals. Twenty‐four lambs were allotted into two feeding group regimens. In the first feeding regimen, lambs (mixed diet, M group, *n* = 12) were housed in well‐ventilated pens with free access to water. The feed was offered in equal amounts at 08:00 and 20:00. The feed intake per lamb was approximately 0.9 kg/day. Composition of the diet is presented in Table 1. The second regimen (pasture grass, PG group, *n* = 12) consisted of allowing lambs to freely graze on wild pasture typical of a semiarid desert steppe (grassland of Inner Mongolia). Pasture‐fed lambs received no supplementation at pasture, which was maintained at a leafy stage and offered ad libitum. The feed intake per lamb was approximately 1.5 kg/day. The dominant grass species were mainly *Achnatherum splendens*, *Artemisia frigida*, *Caragana intermedia*, *Ceratoides latens*, *Cenchrus echinatus Linn*, *Cleistogenes songorica*, *Medicago sativa*, *Suaeda glauca*, and *Stipa capillata*. The nutritional composition of each regimen is shown in Table 2. Lambs were weighted every month before feeding in the morning.

**Table 1:** Ingredients and chemical composition (%) of the mixed diets

Ingredients (%)		Chemical composition (%)	
Maize	25.94	Crude protein	12.6
Soybean meal	15.00	Crude Fat	5.07
Corn silage	52.00	Crude Ash	9.0
Wheat bran	5.04	Ca	0.51
Calcium carbonate	0.23	P	0.36
Calcium bicarbonate	0.11	ADF	33.63
Salt	0.68	NDF	49.17
		ME (MJ/kg)	8.85

Abbreviation(s): ADF: acid detergent fiber; ME: metabolizable energy; NDF: neutral detergent fiber.

**Table 2:** Nutrient composition of each pastures (%)

Pasture name	Crude protein	Crude fat	Crude ash	NDF	ADF	ME(MJ/kg)
*Caragana intermedia*	22.03	4.20	5.72	44.55	29.95	8.54
*Medicago sativa*	32.00	2.18	11.70	36.00	23.36	10.86
*Achnatherum splendens*	8.35	2.29	6.05	76.03	48.41	6.07
*Cenchrus echinatus* Linn	17.30	2.98	13.91	44.16	34.5	8.15
*Suaeda glauca*	16.87	3.38	29.88	37.58	21.72	7.80
*Artemisia frigida*	11.73	4.72	11.12	61.05	47.27	7.72
*Stipa capillata*	12.91	4.52	7.40	65.69	39.01	7.55
*Ceratoides latens*	10.87	2.51	4.99	67.8	48.63	5.22
*Cleistogenes songorica*	12.51	3.23	4.88	68.69	35.29	8.56

### 2.2 Animals and meat samples

The average weight of the lambs at 9 months in the PG and M groups was 32.4 ± 2.9 and 34.6 ± 2.4 kg, respectively. All lambs were slaughtered according to the Operating Procedures of Sheep Slaughtering at a commercial abattoir for experimental and other scientific purposes (Inner Mongolia, DB 65/T 2787‐2007, China). After evisceration, the carcasses were split and cooled to 4°C. Three meat samples were taken from the longissimus thoracic (LT) muscle at 11th thoracic vertebra and deep within the tissue. The two samples were frozen at −20°C for fatty acid (FA) and volatile compound determinations. The last meat sample (10 g) was retained to assess antioxidant enzyme activity and immediately frozen at −80°C.

### 2.3 Meat quality

Samples for meat quality assessment were taken from the LT (5th‐10th rib level) of both sides of the carcass. Analyses were performed in triplicate. Crude protein was determined by Kjeldahl method (GB 5009.5‐2016), and ash was determined by combustion at 550°C for 6 hr. Intramuscular fat (IMF) was determined by the Soxhlet method (GB 5009.6‐2016). The pH was determined using a Matthäus (Germany) pH meter at 45 min (pH_45min_) and at 24 hr postslaughter (pH_24_). The meat color was measured by the L* (lightness), a* (redness), and b* (yellowness) values using a colorimeter (Minolta Chroma meter CR‐410; Konica Minolta Sensing Inc.). For measurements of tenderness, samples (50 g) were packed separately in plastic bags and cooked in a 95°C water bath until the core temperature reached 80°C. Samples were then cooled and prepared for the shear force measurements. One core (1.27 cm in diameter) was excised from each sample parallel to the muscle fiber orientation, through the thickest portion of the cooked muscle. Shear force was determined as maximum force (N) perpendicular to the fibers using a meat tenderness analyzer (Model C‐LM3; Harbin) equipped with a Warner–Bratzler shearing device.

### 2.4 Fatty acids

Intramuscular fatty acids were extracted according to the method described by Folch, Lees, and Sloane (1957). Briefly, LT samples (5 g) were ground with a grinder, mixed with an extraction solvent consisting of chloroform/methanol (2:1, v/v), filtered, transferred to separatory funnels, and mixed with a 20% NaCl solution. After separation, the chloroform lipid fraction was concentrated using a rotary evaporator at 40°C. The lipid extracts were methylated with 1 ml hexane and 3 ml 0.5 N methanolic KOH. The fatty acid methyl esters were analyzed by a GC (SHIMADZU model GC‐2014c) equipped with an Rt‐2560 capillary column (length of 100 m, internal diameter of 0.25 mm, film thickness of 0.25 μm; Restek). The oven temperature was set at 120°C for 5 min, and it was increased to 250°C at a rate of 4°C/min for 28 min. Nitrogen was used as carrier gas at flow rate of 1.1 ml/min. The temperature of the injector was set at 250°C with an injection volume of 1 μl. The detector (FID) temperature was set at 280°C. The individual fatty acid peaks were identified by comparing their retention times with mixtures of standard fatty acids (37 component FAME mixture; 18919‐1 AMP; Supelco) run under the same operating conditions. Quantities of fatty acid in muscle were approximated by comparison of their peak areas with that of tridecanoic acid (13:0) as an internal standard, which was obtained from the total ion chromatograms using a response factor of 1. Fatty acid composition was expressed as mg/100 g of meat.

### 2.5 Volatile compounds

Solid‐phase microextraction (SPME) and GC/MS were employed for the analysis of volatile compounds as described by Marušić, Vidaček, Tibor, Petrak, and Medić (2014). LT muscle samples (5 g) were grounded with a grinder and placed into vials (15 ml) tightly capped with PTFE septa. All samples were stirred during the extraction of volatile compounds.

The SPME samples were coated with a 50/30 μm layer of DVB/Carboxen/PDMS (Supelco), heated at 250°C for 30 min, extracted at 50°C for 45 min, injected to the gas chromatograph–mass spectrometer (GC‐MS) system, and desorbed for 4 min. Volatiles were analyzed in triplicate (*n* = 3). Analyses were performed on a Trace 1300 Series GC gas chromatograph fitted with an ISQ mass spectrometer and a Xcalibur ChemStation (Thermo Fisher Scientific). The injection port, which was held at 250°C, was used to thermally desorb volatiles from the SPME fiber onto the front of the capillary column (TR‐5MS 30 m × 0.25 mm; film thickness 0.25 μm; Thermo Fisher Scientific). Helium was used as the carrier gas at a flow rate 1.0 ml/min. The injector was used in the splitless mode. The temperature program was as follows: 40°C retained for 3 min; increased to 150°C at 4°C/min and maintained for 1 min; and then raised to 200°C at 5°C/min, increased to 250°C at 10°C/min, and maintained for 5 min. The transfer line and ion source temperatures were maintained at 250°C. The mass spectra were obtained at 70 eV, and the scan was ranged from m/z 30 to 400 m/z. Volatile compounds were identified by comparison with data of mass spectra from the library database (NIST MS Search 2.0). Quantities of the volatile compounds were calculated by comparison of their peak areas with that of the internal standard (2‐methyl‐3‐heptanone), which was obtained from the total ion chromatograms using a response factor of 1. Volatile compound composition was expressed as μg/kg meat.

### 2.6 Antioxidant activity

Longissimus thoracic samples (1 g) were grounded with a grinder and homogenized with ice‐cold saline (0.85%, 4.5 ml) and centrifuged (4,000 × *g*, 10 min, 4°C). The supernatants were used to assess catalase (CAT), superoxide dismutase (SOD), and glutathione (GPx) activities.

Catalase activity was determined using a hydrogen peroxidase assay kit (A007‐1; Nanjing Jiancheng Bioengineering Institute) according to the manufacturer's instruction. Absorbance readings were performed at 550 nm (Persee TU‐1810; Persee Co. Ltd.). One unit of CAT activity was defined as 1 mg tissue protein decomposed with 1 μmol H_2_O_2_ per second at 37°C. SOD activity was assessed by measuring the content of hydroxylamine using a total superoxide dismutase assay kit according to the manufacturer's instructions (A001‐1; Nanjing Jiancheng Bioengineering Institute). Absorbance readings were performed at 550 nm. One unit of SOD activity was defined as the amount of enzyme in 1 ml of the reaction solution at 50% SOD inhibition at 37°C. GPx activity was measured colorimetrically using a glutathione peroxidase assay kit according to the manufacturer's instructions (A005; Nanjing Jiancheng Bioengineering Institute). Absorbance readings were performed at 412 nm. One unit of GPx activity was defined as the amount of enzyme capable of decomposing 1 μM glutathione per minute at 37°C.

### 2.7 Antioxidant capacity

The antioxidant capacity was evaluated using three indicators: total antioxidant capacity (T‐AOC), cupric‐reducing antioxidant capacity (CUPRAC), and ABTS^+ ^radical‐scavenging ability (RSA; ABTS). T‐AOC was measured using a total antioxidant capacity assay kit (A015‐1; Nanjing Jiancheng Bioengineering Institute). Absorbance readings were taken at 550 nm (Persee TU‐1810; Persee Co. Ltd.). One unit of T‐AOC activity was defined as an increase of 0.01 in absorbance per second at 37°C using 1 mg tissue protein.

ABTS^+^ radical‐scavenging was evaluated by using a method described by Wen et al. (2015). The ABTS (2,2‐azinobis‐(3‐ethylbenzothiazoline 6‐sulfonate)) reaction solution contained 25 ml of 14 mM ABTS and equal volumes of 4.9 mM potassium persulfate solution. The mixture was maintained in the dark for 16 hr to produce ABTS radical cations (ABTS^+^). The ABTS^+^ solution was diluted with phosphate‐buffered saline (pH 7.4) to an absorbance (734 nm) of 0.70 ± 0.02. Supernatants (as previously described) of the muscle extract (50 μl) were mixed with 6 ml ABTS^+^ and equilibrated at 30°C for 6 min. The control consisted of an equal volume of distilled water in lieu of muscle extract. The percent inhibition of ABTS^+^ was calculated as follows:

$$\text{RSA}\%=\frac{A_{0}-A_{1}}{A_{0}}\times 100$$

*A*_0_ represents the absorbance of blank, and *A*_1_ represents the absorbance of the sample.

CUPRAC was determined using a method described by Apak, Güçlü, Özyürek, and Karademir (2004) with some modification. Briefly, supernatants (as previously described) of muscle extract (50 μl) were added to the reaction system. The reaction system included 1 ml cupric chloride (10 mM), 1 ml ammonium acetate (1 M), and 1 ml neocuproine diluted in 96% ethanol (7.5 mM). The reaction solution was equilibrated at room temperature (22–25°C) for 1 hr. The control contained an equal volume of distilled water in lieu of muscle extract. The absorbance was read at 450 nm. Values are reported as ascorbic acid equivalents in mg/g muscle.

### 2.8 Statistical analysis

Twelve lambs were in each group, and every experiment for each lamb was performed with three replications. All data were analyzed using the Statistical Package for the Social Science (SPSS Inc., version 19.0). Analysis of variance (ANOVA) obtained was further analyzed for the comparison of means by least significant difference (LSD) procedures. Mean values and relative standard deviation (RSD) are reported.

## 3 RESULTS AND DISCUSSION

### 3.1 Feeding regimen and meat quality

The chemical compositions, pH value, color, and tenderness of lamb meat are shown in Table 3. The IMF and ash contents in the PG group were significantly lower than those in M group (*p* < 0.05). Higher IMF levels increase the contribution of SFAs to the proportion of total fatty acids (TFAs) (Howes, Bekhit, Burritt, & Campbell, 2015). Intramuscular fat (IMF) affects tenderness and flavor (Watkins, Frank, Singh, Young, & Warner, 2013). The pH_45min_ value of the PG group was significantly lower than that of the M group (*p* < 0.01). Pasture‐grazed animals are less accustomed to narrow space, which leads to preslaughter stress (Pighin et al., 2015). After 24 hr, the pH value decreased to 5.6. Oliveira et al. (2017) reported that an ideal pH value for meat ranges from 5.5 to 5.8. Lactic acid accumulated in the muscle, which reduces the pH value (Pezeshki, Yavarmanesh, Najafi, Abbaszadegan, & Mohebbi, 2017). With respect to color, the present results showed that the L* (*p* < 0.001) and b* (*p* < 0.05) values in the PG group were significantly higher than those of the M group, which agreed with a previous report by Hajji et al. (2016). Moreover, the PG group also had greater shear force values than the M group (*p* < 0.05), which was related to IMF content (Watkins et al., 2013).

**Table 3:** Meat quality of Mongolian lamb under two feeding regimens (pasture grass and mixed diets)

	PG	M	Significance
IMF (g/100 g)	3.390 ± 0.650^a^	4.996 ± 0.790^b^	*
Crude Protein(g/100 g)	20.98 ± 1.53	20.17 ± 0.67	NS
Ash(g/100 g)	0.872 ± 0.097^a^	0.987 ± 0.066^b^	*
pH_45min_value	6.373 ± 0.172	6.746 ± 0.186	**
pH_24_value	5.640 ± 0.074	5.596 ± 0.049	NS
L*	25.21 ± 1.24^b^	23.01 ± 1.48^a^	*
a*	17.31 ± 3.54	16.58 ± 1.83	NS
b*	8.62 ± 2.49^b^	4.01 ± 0.67^a^	***
Shear force (N)	41.38 ± 5.42^b^	32.94 ± 4.23^a^	*

Abbreviation(s): IMF, Intramuscular fat; M, mixed diet; NS, nonsignificant; PG, pasture grass.

^a,b^Significant differences between feeding regimens (*p* < 0.05).

* *p* < 0.05.

### 3.2 Feeding regimen and fatty acids

In total, 19 fatty acids were identified and quantified in Mongolian lamb LT muscle (Table 4). Palmitic acid (C16:0) and stearic acid (C18:0) were the dominant SFAs. MUFAs were mainly composed of oleic acid (C18:1n9c), while the PUFA fraction largely consisted of linoleic (C18:2n6c) and arachidonic (C20:4n6) acids.

**Table 4:** Fatty acids of meat from Mongolian lamb under two feeding regimens (pasture grass and mixed diets)

	(mg/100 g)	PG	M	Significance
SFA	C12:0	2.03 ± 0.30	2.42 ± 0.37	NS
	C14:0	26.65 ± 2.86^b^	21.50 ± 3.12^a^	*
	C15:0	4.54 ± 0.34	4.06 ± 0.77	NS
	C16:0	348.92 ± 37.62	381.47 ± 41.63	NS
	C17:0	15.50 ± 2.12	14.29 ± 1.76	NS
	C18:0	288.55 ± 22.85	309.94 ± 27.16	NS
	C24:0	1.95 ± 0.238^a^	4.65 ± 0.302^b^	*
MUFA	C15:1	3.70 ± 0.71	3.22 ± 0.41	NS
	C16:1	25.43 ± 2.77	30.25 ± 5.58	NS
	C17:1	6.69 ± 0.75^a^	12.57 ± 1.84^b^	*
	C18:1n9t	20.87 ± 3.29	22.73 ± 8.05	NS
	C18:1n9c	533.29 ± 42.92	600.97 ± 45.59	NS
	C20:1	6.92 ± 1.07^b^	3.08 ± 0.65^a^	*
PUFA	C18:2n6t	3.204 ± 0.57	4.04 ± 1.00	NS
	C18:2n6c	219.56 ± 29.84	200.88 ± 51.03	NS
	C18:3n6	2.15 ± 0.53	2.54 ± 0.27	NS
	C18:3n3	8.60 ± 1.42^b^	5.46 ± 0.83^a^	*
	C20:4n6	174.26 ± 25.34	151.82 ± 33.21	NS
	C22:3n6	10.57 ± 1.61^b^	7.71 ± 1.12^a^	*
Sums	SFA	683.15 ± 64.52	734.42 ± 70.12	NS
	MUFA	559.13 ± 46.51	624.96 ± 50.23	NS
	PUFA	410.67 ± 38.13	376.82 ± 56.13	NS

Note

Values are expressed as mean and standard deviation (*n* = 12).

Abbreviation(s): M, mixed diet; NS, nonsignificant; PG, pasture grass.

^a,b^Significant differences between feeding regimens (*p* < 0.05).

* *p* < 0.05,

** *p* < 0.01;

*** *p* < 0.001.

Differences in the levels of muscle C18:0 and C16:0 between feeding regimens were not significant (*p* > 0.05). Oleic acid (600/533 mg/100 g) was the most abundant MUFA. In addition, no significant differences between the major PUFAs, namely, linoleic and arachidonic acids, were observed. In contrast, muscle from the PG group had a significant (*p* < 0.05) increase in C18:3n‐3 fatty acids. Compared to M group muscles, the total levels of SFAs and MUFAs in PG group muscles were lower, but the PUFA level was higher in PG group muscles. These results were in consistent with previously reported studies (Elaffifi, Bouderoua, & Mourot, 2014 and Duckett et al., 2013).

In the present study, the levels of SFAs and MUFAs in the muscle of M lambs were higher than in PG lambs, indicating that feeding regimens exert a nutritional advantage (Howes et al., 2015). Biohydrogenation in the rumen results in a decrease in muscle PUFAs. Mixed diets, such as those fed to housed animals, are normally rich in saturated and monounsaturated fatty acids. Biohydrogenation in the rumen reduces UFAs to SFAs by microbial lipases to hydrolyzing ester linkages to free a carboxyl group. Thus, mixed diets of lamb meat should deposit more saturated fatty acids than grazed lamb meat. The PUFA content in muscle from the PG group was higher than that in muscle from the M group, which may be due to the nature of the forage (Dierking, Kallenbach, & Grün, 2010; Lourenco, Van, Vlaeminck, De, & Fievez, 2008; Ponnampalam, Burnett, Norng, Warner, & Jacobs, 2012). Pastures are naturally high in PUFAs, especially linolenic acid. Moreover, active ingredients (condensed tannins) in forage effectively reduce the activity of ruminal bacterial and inhibit rumen biohydrogenation, which increases the proportion of PUFAs in animal tissues (Hajji et al., 2016). Tannins can cause a reduction in *Butyrivibrio proteoclasticus*, which convert vaccenic acid to stearic acid, thereby potentially resulting in higher levels of PUFAs in the muscle of PG lambs (Faria et al., 2012; Lobón et al., 2017; Ponnampalam et al., 2012).

As indicated in the present study, the muscle content of n‐3 fatty acids (α‐linolenic acid) was higher in PG lambs, resulting in a concomitant increase in n‐6: n‐3 ratios (Rochfort, Parker, & Dunshea, 2008). α‐Linolenic acid (C18:3n3), an omega‐3 fatty acid, is known to produce EPA and DHA, which are both beneficial in cancer prevention and immune response (Raes, Sde, & Demeyer, 2004). Most forage plants are naturally high in linolenic acid, which is reflected in muscle tissue. Aurousseau, Bauchart, Calichon, Micol, and Priolo (2004) reported that α‐linolenic acid levels in PG lamb muscle are up to twice the levels in M lambs muscle. This difference is ostensibly due to diets rich in C18:3 fatty acids and antioxidants. Antioxidants include carotenes and tocopherols, which occur naturally in forage plants (Resconi et al., 2010). Furthermore, the effects of fatty acids on meat flavor are due to the production of volatile compounds, especially unsaturated fatty acids (Wojtasik‐Kalinowska et al., 2016).

### 3.3 Feeding regimen and volatile compounds

As shown in Table 5, aldehydes were the dominant volatile compounds. The thresholds and concentrations of these compounds affect aroma. In addition, many aldehydes have a low threshold value. The concentrations of hexanal, heptanal, octanal, nonanal, and benzaldehyde were high and considered the main volatile compounds in lamb. Elmore et al. (2005) also reported that the hexanal concentration was highest in lamb meat. Compared with M lambs, the concentrations of hexanal and nonanal in PG lambs were significantly lower (*p* < 0.01), while concentrations of benzaldehyde were higher (*p* < 0.01). A high content of aldehydes in lamb affects meat aroma. In 2005, Elmore et al. reported that hexanal is an indicator of lipid oxidation as it originated from the oxidation of linoleic acid. Meinert, Andersen, Bredie, Bjergegaard, and Aaslyng (2007) reported that high levels of octanal and nonanal in lamb meat are due to oleic acid oxidation. These reports were consistent with the present experimental results. Lipid oxidation directly affects meat quality, included flavor, color, and meat safety (Karabagias, 2018). Overall, muscle aldehyde levels in the M lambs were higher than those in PG lambs and may be related to fatty acid levels. In this respect, The PUFA content of PG lambs was higher than M lambs, which was easily oxidized. However, oxidized aldehydes in PG lambs were lower than those in M lambs, which may have been attributed to the increased antioxidant capacity in muscle of grazed sheep, thereby effectively inhibiting oxidation reactions (Gobert et al., 2010; Muela, Alonso, Campo, Sañudo, & Beltrán, 2014; Petron et al., 2007).

**Table 5:** Volatile compounds of meat from Mongolian lamb under two feeding regimens (pasture grass and mixed diets)

	Compound (μg/1 kg)	PG	M	Significance
Aldehydes	Pentanal	17.14 ± 2.91^b^	11.06 ± 2.38^a^	**
	Hexanal	626.82 ± 66.68^a^	995.41 ± 220.82^b^	**
	Heptanal	69.17 ± 11.40	82.99 ± 27.66	NS
	Octanal	57.61 ± 7.68	46.67 ± 13.03	NS
	2‐Heptenal,(E)‐	ND	4.65 ± 1.65	ND
	Nonanal	133.39 ± 29.53^a^	202.72 ± 54.11^b^	**
	2‐Octenal, (E)‐	3.70 ± 0.93^a^	14.81 ± 5.74^b^	***
	2,4‐Heptadienal, (E,E)‐	2.25 ± 0.26^b^	1.27 ± 0.42^a^	**
	Decanal	2.95 ± 0.50	3.41 ± 1.04	NS
	Benzaldehyde	30.57 ± 7.12^b^	18.59 ± 6.03^a^	**
	2‐Nonenal, (E)‐	6.50 ± 0.65^a^	16.18 ± 5.53^b^	***
	2‐Decenal, (E)‐	3.41 ± 0.67^a^	4.70 ± 0.89^b^	**
	2‐Undecenal, E‐	2.46 ± 0.49^a^	3.58 ± 0.33^b^	***
	2,4‐Dodecadienal, (E,E)‐	3.18 ± 0.88^a^	6.38 ± 2.07^b^	**
	Tetradecanal	2.73 ± 0.55^a^	4.21 ± 0.90^b^	**
	Hexadecanal	6.81 ± 1.81	9.38 ± 2.76	NS
Alcohols	1‐Pentanol	37.30 ± 8.78^b^	22.61 ± 8.55^a^	**
	1‐Hexanol	34.58 ± 7.25^b^	5.03 ± 1.84^a^	***
	1‐Octen−3‐ol	67.30 ± 9.31	75.60 ± 25.43	NS
	1‐Heptanol	14.64 ± 2.99^b^	5.13 ± 1.56^a^	***
	1‐Hexanol, 2‐ethyl‐	4.64 ± 0.78^b^	3.12 ± 2.17^a^	***
	1‐Octanol	27.30 ± 3.41^b^	12.66 ± 3.35^a^	***
	2‐Octen−1‐ol, (E)‐	10.05 ± 2.43	10.30 ± 3.00	NS
	1‐Dodecen−3‐ol	1.58 ± 0.23	1.32 ± 0.17	NS
	Benzyl alcohol	4.24 ± 1.15	5.96 ± 2.65	NS
Ketones	2,3‐Octanedione	122.06 ± 29.22^a^	178.55 ± 30.84^b^	**
	4‐Dodecanone	4.35 ± 1.37	3.79 ± 1.29	NS
Acids	Butanoic acid	2.43 ± 0.48	4.09 ± 1.28	NS
	Hexanoic acid	3.53 ± 0.90	5.61 ± 1.34	**
Hydrocarbons	Ethylbenzene	2.95 ± 0.93	4.96 ± 0.81	NS
	Tridecane	4.15 ± 1.08	ND	ND
	Hexadecane	8.83 ± 1.41^b^	4.16 ± 0.92^a^	***
	p‐Xylene	3.08 ± 0.61	ND	ND
	Tridecane, 3‐methyl‐	6.06 ± 1.11	ND	ND

Note

Values are expressed as mean and standard deviation (*n* = 12).

Abbreviation(s): M, mixed diet; ND, not detected; NS, nonsignificant; PG, pasture grass.

^a,b^Significant differences between feeding regimens (*p* < 0.05).

* *p* < 0.05;

** *p* < 0.01;

*** *p* < 0.001.

In both feeding regimens, nine alcohol‐based compounds from LT muscle were identified including pentanol, hexanol, 1‐octen‐3‐ol, and octanol. These compounds may influence the flavor of lamb meat. Pentanol (*p* < 0.01), hexanol (*p* < 0.001), and octanol (*p* < 0.001) levels in PG lambs were significantly higher than in M lambs. However, no significant differences in the concentration of unsaturated alcohols including 1‐octen‐3‐ol were observed between the feeding regimens (*p* > 0.05). Many alcohols have relatively high threshold values and exert additive effects on meat flavor. Alcohols such as 1‐octen‐3‐ol produce a mushroom, rust‐like odor and have lower threshold values, resulting from the oxidation of linoleic and arachidonic acids (Mariutti & Bragagnolo, 2017). Furthermore, 1‐octen‐3‐ol, which was the predominant alcohol in the present study, is known to contribute to meat flavors (Dirinck, Opstaele, & Vandendriessche, 1997).

High concentrations of ketones, including 2,3‐octanedione, which exerts an earthy, dill‐like aroma, were also detected in lamb meat. The PG group showed higher levels of 2,3‐octanedione compared with the M group (*p* < 0.01) and was consistent with previous reports (Serrano, Cornu, Kondjoyan, Agabriel, & Micol, 2011). Ketones can be produced from lipid autoxidation and microbiological metabolism (Martin, Antequera, Muriel, Perez‐Palacios, & Ruiz, 2009). Previous reports have indicated 2,3‐octanedione as a tracer of pasture‐feeding (Vasta & Priolo, 2006). However, the present study found that the concentration of 2,3‐octanedione in the PG group was lower than that in the M group. Moreover, 2,3‐octanedione originates from the oxidation of linoleic acid, which may be due to the higher oxidation of linoleic acid in M group.

Short chain fatty acids are associated with a goaty odor. The acid compounds included butanoic and hexanoic acids (Table 5). Due to the high threshold value and low concentration of hydrocarbons, those contributed poorly to lamb flavor. With respect to hydrocarbons, alkanes with fewer than 10 carbon atoms are mainly derived from fatty acid oxidation (Mottram, 1998), while those with longer chains are owed to feeding and accumulation in animal depots. Hydrocarbon compounds with a high odor threshold provide little contribution to flavor. The high hydrocarbon content can reflect the relatively high content of unsaturated fatty acids in PG lamb.

### 3.4 Feeding regimen on antioxidant properties

The antioxidant enzyme activities of LT muscle from Mongolian lamb are presented in Table 6. The CAT and GPx activities in the LT of PG lambs were higher than those in M lambs (*p* < 0.001). However, no significant differences were observed between the feeding regimens with respect to SOD. Santé‐Lhoutellier, Engel, and Gatellier (2008) also reported that there were no significant differences in SOD activity between pastured and concentrated lambs.

**Table 6:** Antioxidant status of meat from Mongolian lamb under two feeding regimens (pasture grass and mixed diets)

	PG	M	Significance
SOD (U/mgprot)	79.81 ± 7.27	85.29 ± 8.97	NS
CAT (U/mgprot)	6.06 ± 0.98^b^	3.80 ± 1.05^a^	***
GPx (U/mgprot)	3.08 ± 0.54^b^	1.87 ± 0.45^a^	***
T‐AOC (U/mgprot)	0.62 ± 0.19	0.55 ± 0.14	NS
RSA (%)	28.13 ± 3.04^b^	20.32 ± 3.04^a^	***
CUPRAC (mg/g)	2,950.51 ± 212.77^b^	2,738.36 ± 222.41^a^	*

Note

Values are expressed as mean and standard deviation (*n* = 12).

Abbreviation(s): CUPRAC, cupric‐reducing antioxidant capacity; M, mixed diet; NS, nonsignificant; PG, pasture grass; RSA, radical‐scavenging ability; T‐AOC, total antioxidant capacity.

^a,b^Significant differences between feeding regimens (*p* < 0.05).

* *p* < 0.05;

** *p* < 0.01;

*** *p* < 0.001.

Antioxidant enzymes, including SOD, CAT, and GPx, play a central role in the antioxidant defense system. SOD helps to scavenge superoxide anion by forming hydrogen peroxide, and CAT safely decomposes hydrogen peroxide to H_2_O and O_2_. GPx decomposes both hydrogen peroxide and lipoperoxides that form during lipid oxidation (Gatellier, Mercier, & Renerre, 2004; Santé‐Lhoutellier et al., 2008). The present study indicated that CAT and GPx activities were significantly (*p* < 0.001) different between feeding regimens. Differences in the lamb diet may have contributed to the difference in CAT and GPx activity. The near constant aerobic movement of PG lambs may account for these differences (Gatellier et al., 2004). Antioxidant enzyme activity in the muscle improves and effectively removes free radicals. However, some authors have considered an increase in CAT and GPx activities as a feedback response mechanism to oxidative stress linking to high oxidation degree in concentrated lambs (Petron et al., 2007). Together, these findings indicated that the antioxidant enzymes activity in PG lambs was higher than that in M lambs.

The antioxidant capacity of LT muscle from Mongolian lamb is presented in Table 6. The LT muscle from PG lambs exhibited higher RSA (*p* < 0.001) and CUPRAC (*p* < 0.05) values than M lambs. However, no significant difference in the T‐AOC was observed in LT muscles between feeding regimens. Overall, PG lambs had an increased antioxidant capacity. Importantly, it has been recognized that the feeding pattern affects the antioxidant status in meats (Luciano et al., 2016).

In the present study, muscle from PG lambs had increased antioxidant capacity as determined by RSA and CUPRAC compared with M lambs, which reflected the rich antioxidant compounds (vitamin E) in herbage supplement (Qwele et al., 2013). The present findings were consistent with reports in beef between pasture‐fed and concentrate‐fed patterns (Descalzo et al., 2007). The present findings were in agreement with long‐term grazed lamb fed on herbage containing antioxidant substances, thereby enhancing antioxidant capacity. Antioxidant capacity contributes to oxidative stability in lamb muscles. Natural antioxidants found in herbage improve meat quality, which stabilizes fatty acids to make meat more desirable.

The antioxidant properties (antioxidant enzymes and compounds) in PG lambs may reduce generation of free radicals and inhibit lipid peroxidation. Thus, some aldehydes (off‐flavors) derived from fatty acids in PG lambs were lower than in M lambs, which improved the meat quality of PG lamb.

## 4 CONCLUSIONS

Feeding regimens in Mongolian lambs influenced meat quality in terms of fatty acid and volatile contents. Muscle from M lambs exhibited higher IMF contents (*p* < 0.05), pH_45min _value (*p* < 0.05), and ash contents (*p* < 0.05) than muscle from PG lambs. In contrast, the shear force (*p* < 0.05), L*(*p* < 0.05), and b* (*p* < 0.05) in the M group were lower than those in the PG group. With regard to PUFAs, C22:3n6 and C18:3n3 were higher in PG lambs than in M lambs (*p* < 0.05). The major volatile compounds, including hexanal, nonanal, and 2,3‐octanedione in PG lamb muscle, were significantly lower (*p* < 0.01) than in M lamb muscle. However, 1‐pentanol and 1‐hexanol were higher in PG lambs than in M lambs (*p* < 0.01). Meat from PG lambs had advantages with respect to antioxidant capacity, enzymes, and volatile compounds.

**Highlights**

- Meat obtained from lambs fed mixed diets (M) contained higher levels of IMF than meat obtained from lambs fed pasture grass (PG).
- M lamb muscle contained higher levels of aldehydes and ketones than PG lamb muscle.
- PG lambs showed a better antioxidant capacity than M lambs.

## CONFLICT OF INTEREST

The authors have declared that they have no conflicts of interest in this work.

## ETHICAL APPROVAL

The animal experiments were approved by the Committee of Animal Experimentation and were performed under the institutional guidelines for animal experiments of the College of Animal Science, Inner Mongolian Agricultural University, China. The experiment was performed according to recommendations proposed by the European Commission (1997) to minimize the suffering of animals.
